# The Contribution of Phospholipase A_2_ and Metalloproteinases to the Synergistic Action of Viper Venom on the Bioenergetic Profile of Vero Cells

**DOI:** 10.3390/toxins14110724

**Published:** 2022-10-23

**Authors:** Naira Ayvazyan, Gevorg Ghukasyan, Lusine Ghulikyan, Gayane Kirakosyan, Gohar Sevoyan, Armen Voskanyan, Zaruhi Karabekyan

**Affiliations:** Orbeli Institute of Physiology, Orbely str. 22, Yerevan 0028, Armenia

**Keywords:** snake venom, phospholipase A_2_, metalloproteinases, oxygen consumption, extracellular acidification, chemiluminescence

## Abstract

Increasing concern about the use of animal models has stimulated the development of in vitro cell culture models for analysis of the biological effects of snake venoms. However, the complexity of animal venoms and the extreme synergy of the venom components during envenomation calls for critical review and analysis. The epithelium is a primary target for injected viper venom’s toxic substances, and therefore, is a focus in modern toxinology. We used the Vero epithelial cell line as a model to compare the actions of a crude *Macrovipera lebetina obtusa* (Levantine viper) venom with the actions of the same venom with two key enzymatic components inhibited (specifically, phospholipase A2 (PLA2) and metalloproteinases) in the bioenergetic cellular response, i.e., oxygen uptake and reactive oxygen species generation. In addition to the rate of free-radical oxidation and lipid peroxidation, we measured real-time mitochondrial respiration (based on the oxygen consumption rate) and glycolysis (based on the extracellular acidification rate) using a Seahorse analyzer. Our data show that viper venom drives an increase in both glycolysis and respiration in Vero cells, while the blockage of PLA2 or/and metalloproteinases affects only the rates of the oxidative phosphorylation. PLA2-blocking in venom also increases cytotoxic activity and the overproduction of reactive oxygen species. These data show that certain components of the venom may have a different effect within the venom cocktail other than the purified enzymes due to the synergy of the venom components.

## 1. Introduction

The immense diversity of active molecules found in biological toxins surpasses human-made synthetic compounds. Operating over hundreds of millions of years, natural selection has developed and fine-tuned an incredible assortment of biomolecules, offering an infinite toolkit for drug-discovery programs. Over countless years of natural evolution, biological venoms have been developed and have obtained unprecedented potency and selectivity for rapid interference with specific functional processes, with disruptive and devastating effects on a wide range of fundamental biological processes. It should therefore not be surprising that throughout history, biological toxins had numerous applications, from the treatment of arthritis and gastrointestinal ailments, to modern weapons of mass destruction [1,2,3,4,5]. Novel approaches in basic and translational studies have opened entirely new possibilities for the application of natural toxins to elucidate fundamental physiological and neurobiological processes, as well as to the growing effort to decipher of their therapeutic potential [6,7,8,9,10]. Nevertheless, at the present, only a small fraction of toxins and venoms are characterized pharmacologically. 

One of the biggest problems in fully understanding the mechanisms behind the pharmacological action of natural toxins is the extreme synergy of the venom components, which causes the so-called rapid “spreading effect” with strong inflammatory and necrotizing consequences [11,12]. Hence, simple fractionation and studies of separated venom components in regard to its proteomics often raise more questions than answers, and do not allow deciphering of the exact mechanism of venom proteins in concert. One of the ways to solve this puzzle is to suppress key enzymatic components of venom. Here, we address the individual roles of phospholipase A2 and Zn-metalloproteinases, whose functional effects are well characterized in isolation [13,14,15,16,17], within the complex matrix of the whole venom. 

*Macrovipera lebetina obtusa (MLO)* is a representative of the most common venomous snakes in the Caucasus and the surrounding regions. The venom composition of these snakes includes almost forty active compounds [16]. The majority of studies have been devoted to the function of phospholipase A2 (PLA2) and its effect on cellular membrane bilayer [18,19,20]. This enzyme catalyzes the hydrolysis of the sn-2 fatty acid ester bond of sn-3 phosphoglycerides with consequent liberation of free fatty acids and lysophospholipids [21,22]. Being widely distributed in mammals, endogenous PLA2 is not directly toxic and plays an important role in various physiological processes. It has also been investigated in various pathologies such as asthma, rheumatism, osteoarthritis, and psoriasis [23]. In contrast, many PLA2s from snake venoms are among the most aggressive toxic proteins; often these PLA2s play a major role in prey immobilization and killing [15,24]. Most presynaptic myotoxins and neurotoxins of snake venom composition are either inherently PLA2s or contain PLA2s or PLA2-like subunits in their molecules [16,25]. However, only a portion of the toxic activity of MLO venom can be attributed to purified PLA2 [10,16]. Another important role in venom toxicity is currently ascribed to snake venom metalloproteinases (SVMPs), which are the most abundant component of MLO (~32% of its venom proteome) [17,26,27]. SVMPs cause hemorrhage by damaging the blood vessels’ basement membrane, thus weakening the adhesion between endothelial cells. In addition, low-molecular-weight peptides, disintegrins (such as obtustatin and various dimeric disintegrins) also contribute the adverse effects of MLO venom, although the scientific evidence of this subject is contradictory [28,29,30].

The cellular regulation of metabolic pathways is implemented through cellular adaptation to energy-demand changes in stressful conditions during the course of envenomation. Assessment of the metabolic flux is extremely important for the understanding of the cellular processes. Such an assessment can be carried out via the measurement of oxygen consumption and extracellular acidification using Seahorse Extracellular Flux (XF) Analyzer technology [31,32,33,34,35]. Different studies have established that oxygen free radicals can cause the phosphorylation and activation of various signaling proteins [32,36]. Additionally, the interaction of free radicals with polyunsaturated fatty acids can lead to drastic changes in membrane permeability, alterations in lipid–protein binding, and the formation of products of bioactive degradation [37]. Thus, changes in cellular bioenergetics represent an important component of venom–cell interactions and could complement our understanding of the toxicokinetic response for a distinct cell population. 

Unlike many in-depth contributions that have investigated the pharmacology of individual toxins [8,24,38,39,40,41], the present study offers a general perspective on the prevailing synergistic action of the venom cocktail with some inhibited key actors, providing a new tool for investigations for specialists interested in the processes of cellular bioenergetics, as well as toxinologists with a specific interest in snake venom translational therapeutic capacity.

## 2. Results

### 2.1. Macrovipera Lebetina Obtusa Venom Drives an Increase in Both Glycolysis and Mitochondrial Respiration, While the Venom Preincubated with BPB or EDTA Only Impacts Oxidative Phosphorylation, and Not Rate of Glycolysis

Routinely, the conventional assays for metabolic investigations include those for enzyme activities, protein levels, steady-state ATP levels, and concentrations of metabolic substrates such as glucose and lactate. These end-point measurements yield a static view of metabolism, despite it being a dynamic and rapidly changing cellular process. The technology of Agilent Seahorse XF is capable of assessing the real-time kinetic activity (i.e., rates) of ATP production via two main pathways in cells: mitochondrial respiration and glycolysis. Mitochondrial respiration is measured using oxygen consumption rate (OCR) and is a quantitative metric of mitochondrial function via oxidative phosphorylation (OXPHOS). The Extracellular Acidification Rate (ECAR) is an indicator of glycolysis, and the Proton Efflux Rate (PER), which is a derivation of ECAR, could be calculated as a quantitative measurement of glycolytic rate.

**Figure 1:**
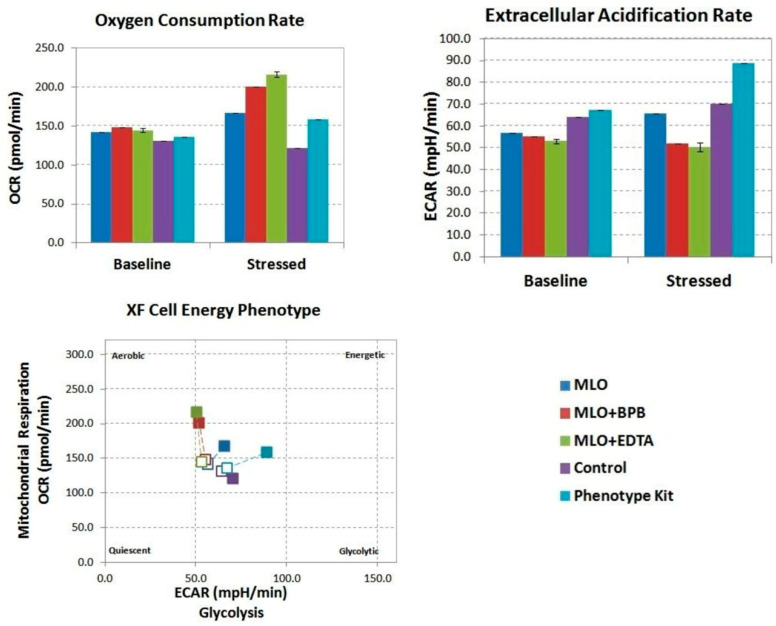
Metabolic phenotype of the bioenergetic state of control and MLO venom-treated Vero cells generated through OCR, and extracellular acidification rate (ECAR) values under normal (herein, basal) conditions and stressed conditions (application of oxidative phosphorylation (OXPHOS) inhibitors of the phenotype stress test kit). Baseline phenotype is OCR and ECAR of cells at starting assay conditions (before the addition of stress factors). The stressed phenotype is OCR and ECAR of cells in the presence of stressor compounds (i.e., under induced energy demand). An increase in the OCR/ECAR ratio compared to the venom-treated cells is indicative of higher OXPHOS activity (experimental compounds injected at 100 μg/mL). Open symbols—baseline, closed symbols—stressed (after injection). MLO—*Macrovipera lebetina obtusa* venom; MLO + BPB—venom treated with bromphenacyl bromide (BPB); MLO + EDTA—venom pre-incubated with ethylenediaminetetraacetic acid (EDTA).

To analyze the data, we compared the OCR and ECAR levels under baseline conditions to their highest rates after compound injection. The data are presented using the Phenotype Test Report Generator built into the Wave software (Agilent Seahorse) (Figure 1). 

**Figure 2:**
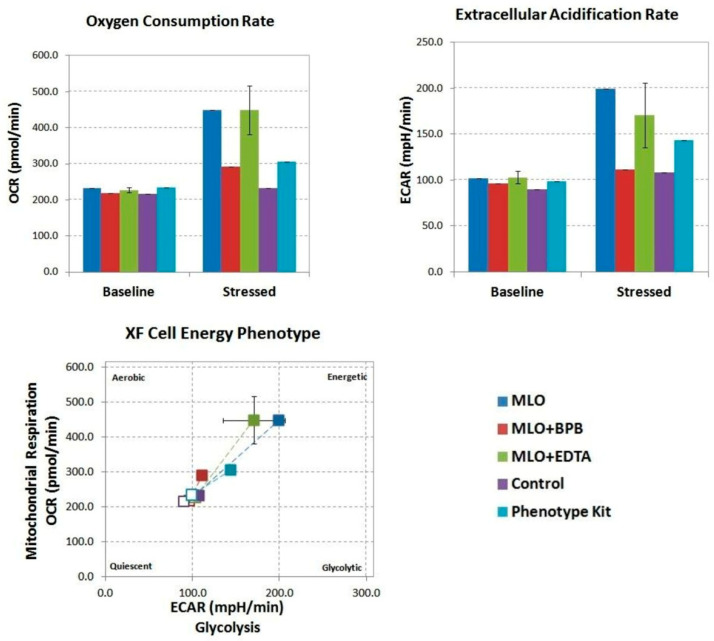
Metabolic phenotype of the bioenergetic state of control and MLO venom-treated Vero cells generated through OCR, and extracellular acidification rate (ECAR) values under normal (herein, basal) conditions and stressed conditions (application of oxidative phosphorylation (OXPHOS) inhibitors of the phenotype stress test kit). Baseline phenotype is OCR and ECAR of cells at starting assay conditions (before the addition of stress factors). The stressed phenotype is OCR and ECAR of cells in the presence of stressor compounds (i.e., under induced energy demand). An increase in the OCR/ECAR ratio compared to the venom-treated cells is indicative of higher OXPHOS activity (experimental compounds injected at 10 μg/mL). Open symbols—baseline, closed symbols—stressed (after injection). MLO—*Macrovipera lebetina obtusa* venom; MLO + BPB—venom treated with bromphenacyl bromide (BPB); MLO + EDTA—venom pre-incubated with ethylenediaminetetraacetic acid (EDTA). We saw a similar correlation at 10 μg/mL for MLO, PSTK, and MLO + BPB; however, it was not as distinct (this figure). Yet, the MLO + EDTA in this case (which is relevant to the sub-lethal physiological concentration) demonstrate more noticeable action in pushing both glycolysis and respiration. We also observed overall higher OCR rates (up to 500 pmol/min), indicating that this concentration of the compounds elicits a more distinctive mitochondrial response, while higher concentrations (i.e., 100 μg/mL) likely start to suppress the overall metabolic response of the cells.

Bromphenacyl bromide (BPB) is a well-known specific inhibitor of PLA2 activity. Ethylenediaminetetraacetic acid disodium (EDTA-Na_2_) is a chelating agent which acts as an effective inhibitor for all metalloproteinases [42,43,44]. The preincubation details of MLO venom with these inhibitors are described in the Section 4. While Vero cells responded similarly to the phenotype test (PSTK) and MLO, both driving an increase in glycolysis and respiration, MLO + BPB and MLO + EDTA exclusively increased oxidative phosphorylation, and not glycolysis (Figure 2).

A lower concentration of the experimental compounds (1 μg/mL and lower) did not seem to elicit a response from the cells (data presented in the Supplementary Materials: Table S3).

### 2.2. During the MTT Test, the BPB-Blocked Venom Demonstrates the Most Cytotoxic Activity Even at Very Low Concentrations

**Figure 3:**
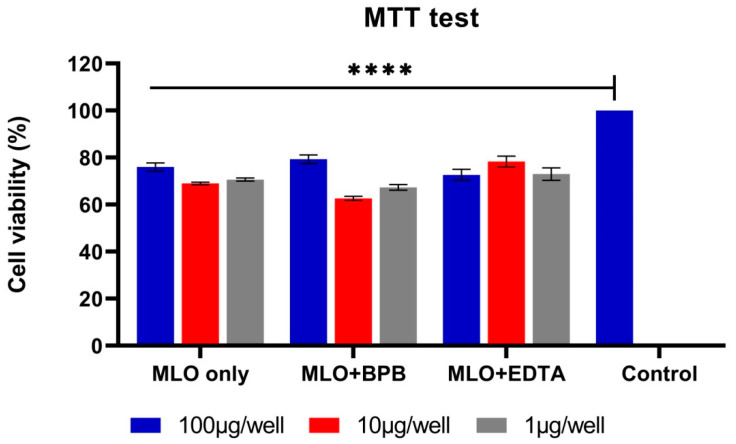
MTT-assessed cell viability of Vero after treatment with different concentrations of MLO venom and the same venom pre-incubated with BPB and EDTA to inhibit phospholipase A2 and metalloproteinase activities (**** *p* < 0.0001 in relation to the control/untreated cells; ANOVA, followed by the Tukey test). MLO—Macrovipera lebetina obtusa venom; MLO + BPB—venom treated with bromphenacyl bromide (BPB); MLO + EDTA—venom pre-incubated with ethylenediaminetetraacetic acid (EDTA).

In view of the synergistic potential of the investigated venom components in Vero cells, the cytotoxic dose–response effect of the whole venom and its PLA2- and Zn-metalloproteinase-inhibited versions were assessed using the MTT assay (Figure 3). The results demonstrated that the most toxic component to the cells was the BPB-blocked venom at a 10 μg/well concentration, although all three solutions of venom induced cell death up to 63–80% of their viability, but not in a dose-dependent manner. The effect of the SVMP-inhibited venom on the viability of cells was not as drastic as the effect of either crude or BPB-blocked venom, but it was still very noticeable and statistically significant. To check their possible cytotoxicity in the Vero cells, the EDTA and BPB solutions without the venom were used as negative controls. Interestingly, the addition of the EDTA disodium even increased the viability of Vero cells (3%), while the BPB alone could also be slightly cytotoxic (data presented in the Supplementary Materials, Figure S1). 

### 2.3. ROS Overproduction Is Noticed Both in the BPB-Blocked and Crude Venom, While after Switching off the SVMPs, the Venom Became an Antioxidant

**Figure 4:**
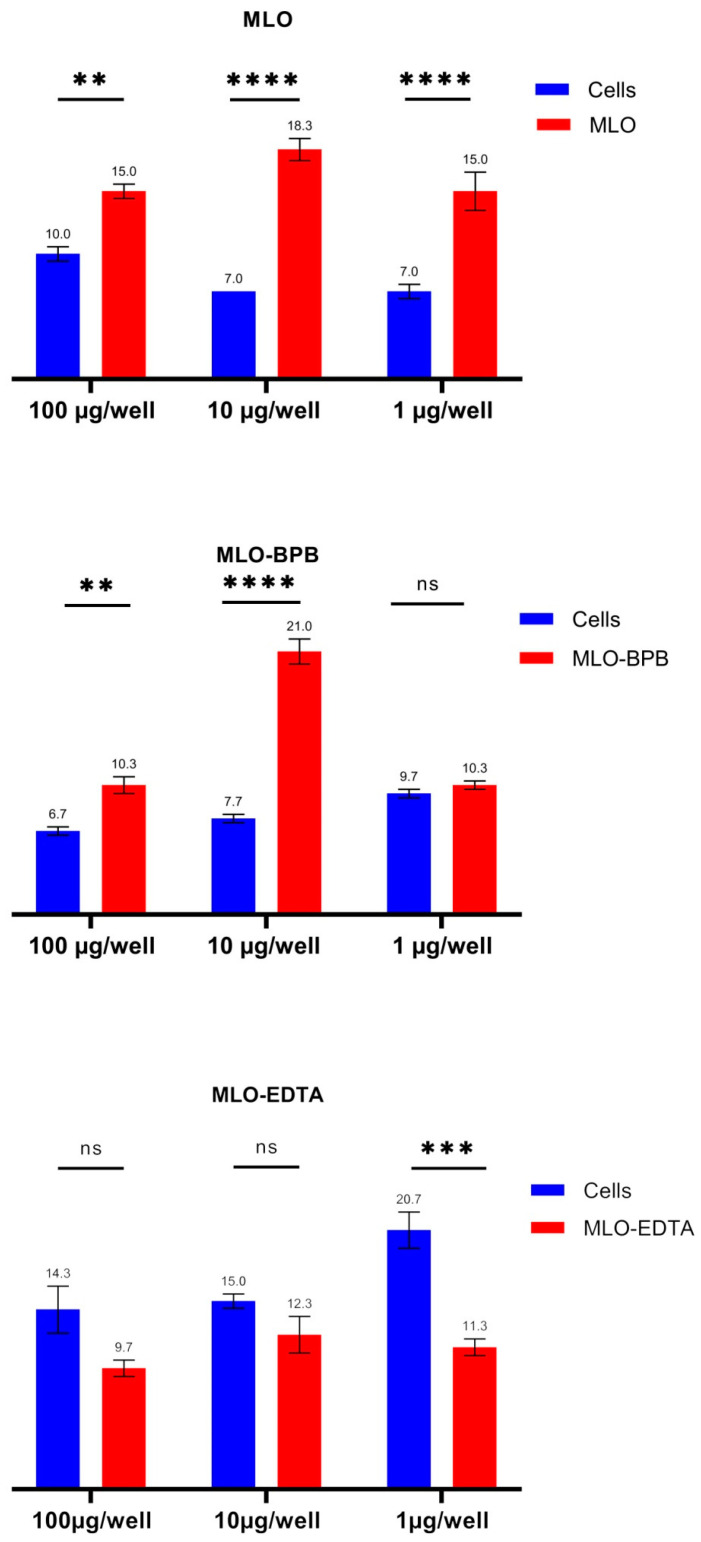
The ROS production levels of Vero cells during the course of intoxication by the *Macrovipera lebetina obtusa* venom and phospholipase A2 and metalloproteinase activities blocked in the venom assays. Data presented as spontaneous chemiluminescence intensity measured in a real-time mode as the impulse/sec for three different concentrations of venom per well (two-way ANOVA test). MLO—Macrovipera lebetina obtusa venom; MLO-BPB—venom treated with bromphenacyl bromide (BPB); MLO-EDTA—venom pre-incubated with ethylenediaminetetraacetic acid (EDTA) (** *p* ≤ 0.01; *** *p* ≤ 0.001; **** *p* ≤ 0.0001).

Noticeable increases in the means of the ChL counts were observed in all samples during the course of MLO and MLO-BPB venom in vitro processing (Figure 4), but again, the most remarkable changes were detected at a 10 μg/well concentration, yet even at the lowest concentration, the increase in ROS production for crude venom was statistically significant in comparison to that of the control. As a control, here, we used untreated cells, while the negative controls (BPB and EDTA solutions, as well as venom solutions) demonstrated spontaneous ChL intensity very close to that of the equipment noise values: 5–6 impulse/sec.

**Figure 5:**
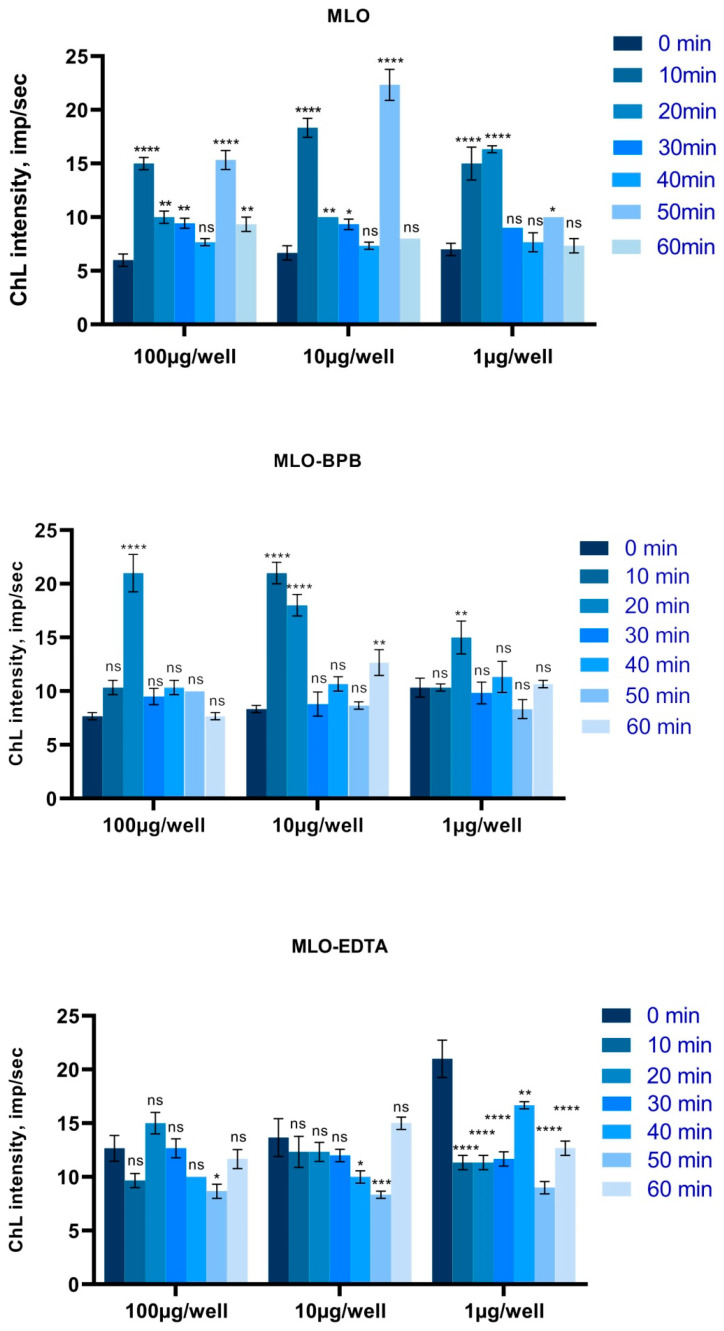
Kinetic changes in the spontaneous chemiluminescence levels of Vero cells during the course of real-time mode processing with *Macrovipera lebetina obtusa* venom and phospholipase A2 (MLO-BPB) and metalloproteinase (MLO-EDTA) activities blocked venom assays (two-way ANOVA test) (* *p* ≤ 0.05; ** *p* ≤ 0.01; *** *p* ≤ 0.001; **** *p* ≤ 0.0001).

The results of the free-radical scavenging effect of the snake venom extract in the in vitro assay with blocked metalloproteinase activity, in contrast, showed significant in vitro antioxidant activity. This interesting observation suggests without doubt that SVMPs are the pro-oxidative components of venom, while PLA2s in venom composition, as well as some other components such as disintegrins and/or serine proteinases, have a radioprotective role and could act as free-radical traps [9,19]. The kinetic line of the ROS generation of Vero cells has a typical periodic curve, which is obviously a concern of respiration (Figure 5).

### 2.4. Lipid Peroxidation Processes Are Also Remarkably Higher in PLA_2_-Inhibited Venom

**Figure 6:**
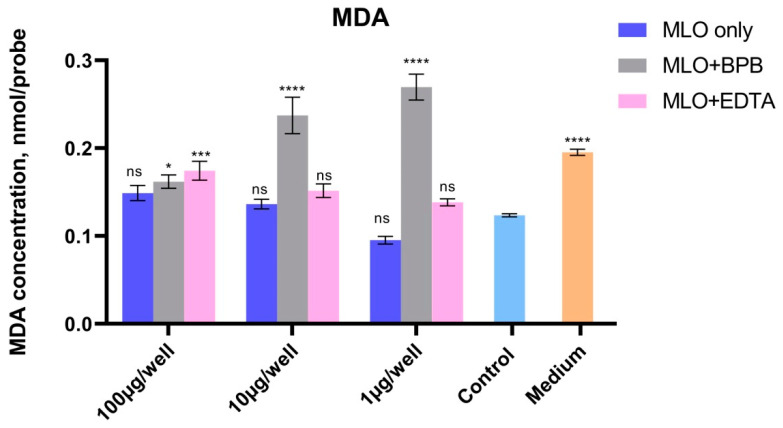
The concentration of malonic dialdehyde as a criterion of the level of lipid peroxidation in Vero cells after processing with *Macrovipera lebetina obtusa* venom and phospholipase A2 (MLO + BPB) and metalloproteinase (MLO + EDTA) activities blocked venom assays. Vero cells were incubated with each assay at 37 °C for a period of 10 min. MDA concentration was measured at 532 nm after reaction with thiobarbituric acid (TBA) at 100 °C (* *p* ≤ 0.05; *** *p* ≤ 0.001; **** *p* ≤ 0.0001).

When we analyzed the accumulation of MDA in untreated Vero cell cultures and after processing using crude MLO venom and using the same venom with inhibited phospholipase A2 and metalloproteinase activities, we observed remarkable divergence of the MDA concentration in all assays in a dose-dependent manner (Figure 6). The suppression of phospholipase activity led to an enormous increase in the lipid’s free-radical oxidation, which was almost two times higher than the level of lipid peroxidation of the untreated cells in the case of the lowest dose of the venom, but less at the 10 µg/well concentration, and almost insignificant for the highest concentration of the BPB-blocked venom. It is important to mention that while venom solutions (MLO, MLO-BPB, and MLO-EDTA) have no level of lipid peroxidation, the level of the MDA accumulation in the cell culture medium could be artificially increased due to the high content of sugars.

Under EDTA-preincubated venom action, MDA formation was slightly activated compared with the untreated cells in a dose-dependent manner, which indicates intensification of the peroxidative processes. According to the literature data, the clinical manifestations of viper venom’s influence are different for different tissues. For mammals, which are the most-studied class of animal, a sublethal dose of MLO venom had a radioprotective effect [45,46]. Our data suggest that phospholipase A2s are the main components that are responsible for such a protective effect.

## 3. Discussion

The venom proteome of the *Macrovipera lebetina obtusa* is comprised of a complex arsenal of peptides and proteins with unique pharmacological properties, which can seriously alter cellular metabolism irrespective of the cell type or the metabolic background [18]. This complex influence could be further complicated due to the extreme synergy of the venom components’ actions. A detailed analysis of the biochemistry of MLO venom demonstrated that, among venom proteins, snake venom Zn-dependent metalloproteinases of the PI and PIII classes [18] represent the most abundantly expressed gene family (~32% of the total venom proteome). Other major toxin families are phospholipase A2 (PLA2, 14.6%), snake venom serine proteinases (SVSP, 14.9%), and C-type lectin-like proteins (14.8%), with similar relative abundance. The remaining 23.7% of the venom proteome is comprised of six toxin classes, none of which exceed 5% relative abundance; still, their impact in complex pharmacological reactions on pray organisms should not be underestimated. The overall toxic effect of this cocktail is quite severe and causes alterations in all the main metabolic processes of cells.

As a chain of metabolic reactions that takes place in the cells to convert chemical energy from nutrients or oxygen molecules into adenosine triphosphate (ATP), cellular respiration could be considered one of the key processes in fueling cellular activity through the release of chemical energy [47,48]. This is one of the first studies with such a comprehensive metabolic extracellular flux analysis of epithelial cells treated with crude snake venom, with and without its specifically inhibited derivates.

Glucose and glutamine are the main sources of carbon for mammalian cells and they are essential for the generation of ATP and various biosynthetic reactions [49]. The oxidation of glucose during the course of glycolysis with pyruvate and ATP generation leads to the involvement of pyruvate in the tricarboxylic acid (TCA) cycle as acetyl coenzyme A (acetyl-CoA). The TCA cycle is a key factor in the oxidation of nutrients to support electron flow along the electron transport chain, and hence, in the production of ATP during course of oxidative phosphorylation (OXPHOS). Meanwhile, during course of oxidative glutaminolysis, glutamine can anaplerotically replenish the TCA cycle to enable the continued generation of reducing equivalents (NADH and FADH2) and intermediates of the TCA cycle [50].

With these constraints, the oxygen consumption rate is a direct and quantitative measure of the mitochondrial electron transport rate. Another equally accessible measurement of metabolic activity is extracellular acidification, a major component of which is the glycolytic production of lactate [35,51]. Under physiological conditions (namely, pH around 7), glucose is uncharged, while lactate (pKa, 3.86) exists as the carboxylate anion. Glucose–lactate conversion under neutral pH conditions leads to the protons’ release, which consequently leads to acidification of the medium, so the rate of the latter (extracellular acidification) could be used as a direct quantitative measure of the rate of glycolysis [49,51,52,53,54].

The data in this study underline PLA2 as one of the key factors in the snake venom complex’s influence on cellular metabolism. Venom injection induces a metabolic shift of Vero cells to a higher energy level; however, if MLO venom causes the activation of both glycolysis and mitochondrial respiration, the switching off of the phospholipase and metalloproteinase activities of the same venom exclusively intensifies oxidative phosphorylation, and not glycolysis. It is important to mention that the lowest concentrations of venoms (both crude and with added inhibitors) did not elicit a metabolic response from the cells, despite their effect on cell viability. The latter declined to 80%, even after the lowest concentrations of venom were used.

To date, there is no information about the exact concentration of venom reaching the heart or any other tissue/organ after a snake bite. So, we can only suggest that the estimated concentrations of venom used in this study are physiologically relevant. Hence, we observed the impact of the venom from a 1µg/mL concentration and up to 100 µg/mL in a dose-dependent manner for the numerous lines of cell cultures (data not presented), and empirically chose the discussed ones as physiologically relevant frontiers. The concentration of 1 µg/mL used in this study is the concentration which is capable of inducing minor metabolic changes, while the concentrations of the experimental compounds injected at 100 and 10 µg/mL are relevant to the lethal and sub-lethal physiological doses for the venom based on previous experimental findings [55,56,57].

The metabolic functions of glucose and glutamine are overlapping [58,59]. This is why both nutrients are required for cell viability. The venom-associated intensification of glycolysis could also be a compensatory mechanism for the generation of reactive oxygen species through the higher rate of mitochondrial respiration [60]. The direct measurement of ROS generation in real-time mode shows Zn-dependent metalloproteinase activity as a main culprit of oxidative stress induction, while, surprisingly, the inhibition of marked activity leads to the antioxidant effect of venom on the free-radical processes. Free radicals, in general, are highly reactive and extremely short-lived, which is why they are elusive and hard to detect. Therefore, we often have to search for end-products or by-products of radical-induced reactions, examining the reaction “path” of the radicals in order to confirm their production. The only direct way to detect and measure free radicals in real-time mode is ChL analysis; so, the complex approach is the best way to study reactions that generate free radicals and cause oxidative damage. It is highly likely that for this antioxidant phenomenon, PLA2 is not the only one responsible, especially if we take into account that disintegrins are cysteine-rich peptides, known as strong antioxidants. 

Our results show some differences between the TBA test and ChL analysis, limited, as we suggest, by the methodical specification of the TBA test; this depends on the participation of di- and polyunsaturated fatty acids exclusively, in the formation of MDA products, but not monounsaturated ones. Nevertheless, during the course of ChL analysis, the product of monounsaturated fatty acids—hydroperoxides—influences the level of the ChL intensity. Thus, as we believe, the specificity of action of the MLO toxins [30,60,61,62], which play a key role in the radioprotection effect, can be assessed by the level of monounsaturated fatty acids of the cell membranes, as well as the membranes of mitochondria. We have previously demonstrated the influence of venom on rat brain lipids, consisting of monounsaturated and saturated fatty acids (mainly neuron and stearic fatty acids) in contrast with other types of lipid [45,63,64].

Therefore, the current study shows that the investigated viper venom leads to an increase in both glycolysis and respiration in normal epithelial cells, while the same venom with blocked PLA2 and metalloproteinases exclusively affects oxidative phosphorylation. The inhibition of the enzymatic PLA2 activity within the venom decreased the metabolic activity at even low concentrations, but enhanced lipid peroxidation and ROS overproduction. 

The most important outcome of this work is the evidence of the synergistic effect of viper venom proteins on the bioenergetic phenotype of epithelial cells, while some of the venom’s key components are individually inhibited. Certainly, there are limitations to studying the mechanisms of action of such a complicated cocktail of potent proteins and enzymes, as there are with any model system. However, these kinds of studies comparing the effect of the whole venom with the effect of the venom cocktail, where some separate protein components are switched off, seems to be very promising for future toxinological investigations; these could lead to a better understanding of both venoms’ pharmacology and the mechanisms of alteration of cellular bioenergetics.

## 4. Materials and Methods

### 4.1. Reagents and Venom

The chemicals were from Sigma. The *macrovipera lebetina obtusa* (MLO) venom was purchased from Latoxan (Portes-lès-Valence, France). The bromphenacyl bromide (BPB) and ethylenediaminetetraacetic acid disodium (EDTA-Na_2_) were from Sigma-Aldrich (Saint Louis, MO, USA). The cell culture reagents and consumables were from Thermo Fisher Scientific (Waltham, MA, USA). The consumables used in the Seahorse XF assays were from Agilent Technologies (Santa Clara, CA, USA).

### 4.2. Preparation of PLA_2_ and/or Metalloproteinases Inhibited Venom

BPB is a specific inhibitor of PLA2s. On the day of the experiment, a 1/10 ratio of the inhibitor to the venom (100 μg BPB/1 mg MLO) was taken and incubated at room temperature for 2 h in phosphate buffer (pH 7.4) with periodic mixing, to allow time for BPB to interact with the PLA_2_ [65].

EDTA-Na_2_ salt was used to inhibit the metalloproteinase of the whole MLO venom. The experiment was designed as described in Kurtovic et al., 2014, with modifications: 100 μg EDTA-Na_2_ was pre-incubated with 1 mg lyophilized crude venom at 37 °C in phosphate buffer (pH 7.4) 2 h prior to the experiment [44].

### 4.3. Analysis of the Oxygen Consumption Rate (OCR) and the Extracellular Acidification Rate (ECAR)

The OCR and ECAR were evaluated using the Seahorse XF Cell Energy Phenotype Stress Test Kit (PSTK: oligomycin with p-trifluoromethoxy carbonyl cyanide phenylhydrazone (FCCP), Agilent Technologies, USA) following the manufacturer’s protocols, and measured using an XFp analyzer (Agilent Technologies) [50]. Briefly, 100 µL of 20,000 Vero cells per well were seeded in Seahorse XFp cell culture plates the day before the measurement and incubated for 24 h. On the day of the assay, the culture medium was replaced with an XF base medium (Agilent Seahorse Technologies) supplemented with 2 mM Gln, 1 mM Pyr, and 10 mM Glc, with an adjusted pH of 7.4. The cells were then incubated at 37 °C without CO_2_ for 1 h and measured using an XFp analyzer. Prior to the measurement, 10 μL of freshly prepared venom (MLO only, MLO + BPB, MLO + EDTA) was added to each well at the following final concentrations of venom: 100 µg/well, 10 µg/well, and 1 µg/well. The preliminary set of measurements were carried out to be sure that BPB and EDTA in the above-mentioned concentrations were not able to make a significant change to the bioenergetic profiles of the Vero cell cultures. Three baseline OCR and ECAR measurements were made, followed by an injection of a “stressor mix” (PSTK (Agilent Seahorse) containing the following final concentrations of two inhibitors: oligomycin (2 M) and p-trifluoromethoxyphenylhydrazone (FCCP; 0.8 M)) as a positive control, and/or a venom cocktail with inhibitors. Six further measurements were taken and data were analyzed using Wave 2.6 software (Agilent Technologies).

### 4.4. Chemiluminescence Analysis and Lipid Peroxidation

Reactive oxygen species (ROS)’ levels were measured using a chemiluminescence (ChL) analyzing system Junior LB 9509 portable tube luminometer (BERTHOLD TECHNOLOGIES, Bad Wildbad, Germany) [66]. Unstable lipid peroxides are easily decomposed to a complex series of compounds, the most abundant of which is malonic dialdehyde (MDA). MDA’s level of tissues was determined via spectrophotometric measurement [45,67] using the TBA test, based on the reaction of a chromogenic reagent, thiobarbituric acid (TBA), with MDA at 100 °C. During the course of this process, two molecules of MDA react with one molecule of TBA to yield a stable three-methin complex dye. The final concentration of MDA was measured at 532 nm using a spectrophotometer (B01-CT-8, “E-ChromTech”, Taiwan).

### 4.5. MTT-Assessed Cytotoxic Effect on Vero Cells

Vero cells (African green monkeys’ kidney epithelial cells; IMB, USA) were cultured in Dulbecco’s modified Eagle medium (DMEM) with 5% *v/v* fetal bovine serum (FBS; Gibco, USA), 100 U/mL penicillin, and 100 μg/mL streptomycin. The metabolic effects of unmanipulated MLO venom and venom partially inhibited by EDTA (to prevent the activity of metalloproteinases) or by BPB (to prevent the activity of phospholipase A) were assessed using two parallel assays: a standard methyl-thiazolyl-tetrazolium (MTT) colorimetric assay and the Seahorse FX analyzer. For the MTT assay, 24 h prior to the assay, Vero cells were plated into microplates at a concentration of 0.2 × 10^6^ cells/well in 100 μL culture medium (tissue culture grade, 96 wells, flat bottom). The cell cultures were incubated for 24 h at 37 °C and 5% CO_2_. After 24 h of incubation, 10 μL of freshly prepared venom (MLO only, MLO + BPB, MLO + EDTA) was added to each well at the following final concentrations of venom: 100 µg/well, 10 µg/well, and 1 µg/well. The control wells were treated identically but did not contain any venom. Cells treated with venoms were incubated for 1 h at 37 °C and 5% CO_2_. After a 1 h incubation of 10 µL of the 12 mM solution, MTT labeling reagent was added to each well and they were incubated for another 2 h. The 12 mM stock solution was prepared by dissolving 5 mg of MTT [3-(4,5-dimethylthiazol-2yl)-2,5-diphenyltetrazolium bromide] in 1 mL of PBS. Finally, 120 μL of the Solubilization solution (1 g of SDS in 10 mL of 0.01 N HCl) was added to each well. The absorbance was measured at a 568 nm wavelength using a HiPo MPP-96 microplate reader (BioSan, Latvia). The recorded data were quantified using the provided Quant Assay software.

### 4.6. Statistics

The MTT viability and Seahorse FX analyzer experiments included three independent cell preparations with all conditions run in triplicate. The Representative Phenotype Test Report Generator images are shown. The mean values for cell viability are expressed as a percentage of vehicle control and were analyzed using ANOVA, followed by the Tukey test. All values are expressed as mean ± SE, with *p* < 0.05 considered statistically significant. Statistical analyses for chemiluminescence assay and lipid peroxidation were performed using two-way ANOVA (GraphPad Prism 8, San Diego, CA, USA).

## Data Availability

No new data were created or analyzed in this study. Data sharing is not applicable to this article.

## Supplementary Materials

The following supporting information can be downloaded at: https://www.mdpi.com/article/10.3390/toxins14110724/s1, Figure S1: MTT negative control; Table S1: SeaHorse measurements for 100 µg/well; Table S2: SeaHorse measurements for 10 µg/well; Table S3: SeaHorse measurements for 1 µg/well.

## Sample Availability

Samples of the compounds are available from the authors.
