# Supplementary material for: The Contribution of Phospholipase A2 and Metalloproteinases to the Synergistic Action of Viper Venom on the Bioenergetic Profile of Vero Cells
Source: Toxins (Basel). 2022 Oct 23;14(11):724. doi: 10.3390/toxins14110724 (PMC9695613; doi:10.3390/toxins14110724)
Supplement: Supplementary file 1 [file toxins-14-00724-s001.zip › Figure S1.pdf]

toxins

Supplementary Materials

The Contribution of Phospholipase A2 and Metalloproteinases to the Synergistic Action of Viper Venom on the Bioenergetic Profile of Vero Cells

Naira Ayvazyan *, Gevorg Ghukasyan, Lusine Ghulikyan, Gayane Kirakosyan, Gohar Sevoyan, Armen Voskanyan and Zaruhi Karabekyan

Orbeli Institute of Physiology, Orbely str. 22, Yerevan 0028, Armenia

* **Correspondence: taipan@ysu.am; Tel.: +374-10-272247**

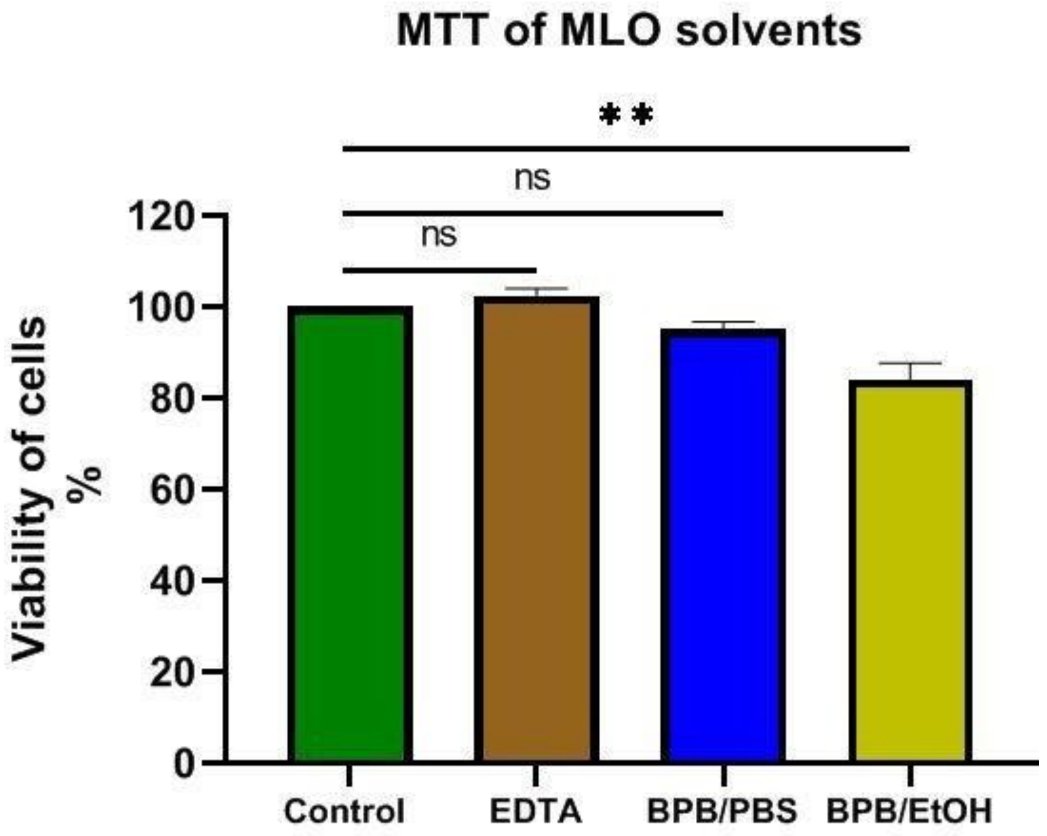

Figure S1. MTT negative control

Negative control: Viability of cells after addition of the BPB and EDTA solutions (the same concentrations used in the experiments). BPB in ethanol wasn't used because of the significant effect on the MTT-test results (** means $p<0.004$).

GraphPad Prism 8:

**** means $p<0.0001$

** means $p<0.004$

* means $P<0.01$ and $p>0.05$ is ns (non significant)
